# Current and Future Geriatrics Education Policy Initiatives

**DOI:** 10.1093/geroni/igaa057.1803

**Published:** 2020-12-16

**Authors:** Marla Berg-Weger, Katherine Bennett

**Affiliations:** 1 Saint Louis University, St. Louis, Missouri, United States; 2 University of Washington, Seattle, Washington, United States

## Abstract

Current and future NAGE policy-related activities will be the focus of this presentation. The Geriatric Academic Career Awards (GACAs), which support the career development of junior faculty clinician educators in geriatrics, were reinstituted by HRSA in 2019 after a 13-year absence. We will discuss the role of this award in the broader context of geriatrics education and GWEPs, how GACA awardees have been integrated into NAGE, and the need for expansion of the GACA program to support both the GWEP and geriatric education pipelines. Areas for future NAGE engagement will be focused on advocacy efforts to support: permanent GWEP reauthorization by Congress; expanding current level of $40.737 million to $51 million to enable HRSA to increase the number of GWEPS to further extend their reach; increasing funding for GACA awardees; and strengthening the synergies between the GACA and GWEP programs to support development of future GWEP leadership.

